# The Effect of Harsh Environmental Conditions on Concrete Plastic Shrinkage Cracks: Case Study Saudi Arabia

**DOI:** 10.3390/ma15238622

**Published:** 2022-12-02

**Authors:** Talal O. Alshammari, Maurizio Guadagnini, Kypros Pilakoutas

**Affiliations:** 1 Department of Civil and Structural Engineering, The University of Sheffield, Sir Frederick Mappin Building, Mappin Street, Sheffield S13JD, UK; 2 Department of Civil and Structural Engineering, College of Engineering, Jouf University, Sakaka 72388, Saudi Arabia

**Keywords:** early age concrete cracking, plastic shrinkage concrete cracks, concrete shrinkage: digital image processing, recycled tire steel fiber, hot weather concreting, Saudi Arabia environmental conditions

## Abstract

Due to climate change and population expansion, concrete structures are progressively being subjected to more extreme environments. As the environment affects plastic shrinkage directly, there is a need to understand the effect of environmental changes on plastic shrinkage cracking. This paper examines the plastic shrinkage crack development parametrically at low, mid, and high drying environmental conditions, corresponding to different environments in three Saudi cities. The effects of water-cement ratios and quantities of recycled tire steel fibers (RTSF) in concrete are also investigated. The different environmental conditions for the plastic shrinkage tests were simulated in a specially designed chamber as per ASTM C1579, 2006. A digital image processing (DIP) technique was used to monitor crack initiation and development. Through the use of the crack reduction ratio (CRR), it was found that 30 kg/m^3^ of RTSF can control plastic shrinkage cracks at low and mid conditions. For the more extreme (high) conditions, the use of 40 kg/m^3^ of RTSF fiber was sufficient to completely eliminate surface plastic shrinkage cracks. This work can help develop more sustainable concrete structures in a wider set of environmental conditions and help mitigate the impact of climate change on concrete infrastructure.

## 1. Introduction

Climate change is creating more extreme environments around the world and is impacting concrete durability. Population expansion is also pushing development in more extreme environments. CO_2_ emissions and high energy consumption (including for cement production) are some of the reasons for extreme heat waves in Middle East countries such as Saudi Arabia as well as Europe, Asia, and North America [1,2]. Many countries, such as Saudi Arabia, where in some parts, air temperature can exceed 50 °C in the summer, are struggling to produce quality concrete in hot weather conditions. Early-age environmental conditions, including air temperature, wind speed, and relative humidity, can affect concrete in its plastic stage adversely by accelerating early-age plastic shrinkage cracks [3].

Long-term concrete cracking is unavoidable, and large openings impact concrete durability [4,5]. In hot and dry areas of the world, high air temperature, wind, and low relative humidity are also known to impact durability [6], as they can cause high plastic and drying shrinkage strains in concrete [7,8,9]. ACI 224R-01 [10] attributes early-age concrete cracks to excessive evaporation due to environmental conditions prior to concrete setting. The earlier concrete cracks develop, the shorter the serviceable life of concrete is expected [11,12]. Plastic shrinkage cracks are the earliest to appear, as they occur two-three hours after casting, prior to setting. Subsequent propagation of plastic shrinkage cracks will allow ingress of water and offensive agents such as chlorides and increase the possibility of concrete deterioration and corrosion of steel rebars [13,14]. Plastic shrinkage cracks not only reduce concrete durability but are also aesthetically undesirable [15].

**Figure 1:**
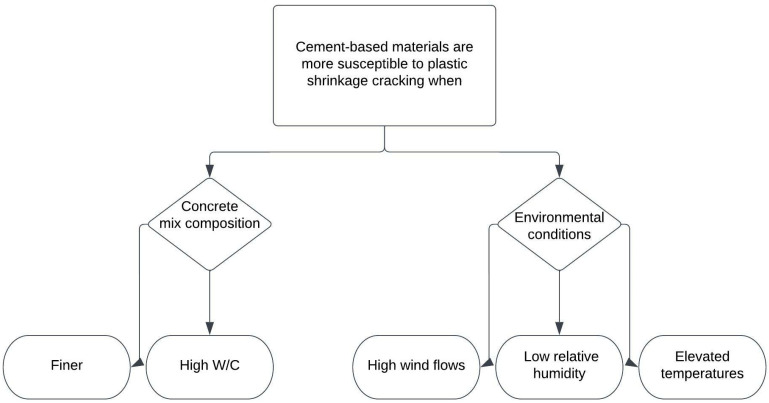
Factors affecting plastic shrinkage cracking [22].

Volume changes in concrete before the hardening of cement-based materials are the main cause of plastic shrinkage strain and cracking [16,17]. Volume loss at the plastic stage is caused by the consolidation of aggregates, bleeding, and evaporation of water. In its plastic state, when undisturbed, the denser solid particles settle and tend to sink down, whilst the lighter-weight materials, such as air and free water, begin to rise to the surface. Air escapes faster, but the escaping water, called bleeding water, escapes slower, and when it reaches the surface, it starts evaporating [18]. When the evaporation rate exceeds the bleeding rate, the concrete surface dries, and at this stage, the possibility of plastic shrinkage cracking increases [19,20,21]. Both environmental conditions and concrete mix composition affect plastic shrinkage, as seen in Figure 1 [22].

Hot weather casting is known to increase plastic shrinkage cracking [23]. It is widely accepted that plastic shrinkage starts when the evaporation rate exceeds the bleeding rate. Several studies reported that environmental conditions such as high air temperature, high wind speed, and low relative humidity have a direct effect on fresh and hardened concrete, as they also accelerate the final set time [24,25].

Ambient conditions influence the water evaporation rate. As air temperature increases, relative humidity sees a corresponding decrease, and thus evaporation rate increases. Higher wind speeds also increase the evaporation rate. When the evaporation rate is less than the bleeding rate, a thin layer of water covers the surface of the concrete, which helps increase the evaporation rate due to the increased exposed area [26].

Eventual drying of the surface leads to a rise in capillary pressure converting it from a mildly compressive to a tensile pressure [27]. When capillary pressure inside the concrete builds up, plastic shrinkage cracking will occur.

According to ASTM C1579 [28], plastic shrinkage cracks occur when the water evaporation rate is equal, or more than, 1.0 kg/m^2^/h. However, Sayahi and Hedlund [26] found that most existing researchers conclude that this value is too high and plastic shrinkage cracks might appear when the evaporation rate is less than 1.0 kg/m^2^/h, especially in hot weather conditions. For example, Almusallam et al. [29] concluded from observations in two studies that the beginning of plastic shrinkage cracking could occur at an evaporation rate between 0.2–0.7 kg/m^2^/h.

To examine the possibility of plastic shrinkage cracking in concrete, ASTM C1579 [28] recommends a set of environmental conditions to be applied: air temperature 36 ± 3 °C, wind speed more than 4.7 m/s, and relative humidity around 30 ± 10%. These environmental conditions were selected based on past experimental work [28]. However, Al-Gahtani et al. [30], working in the eastern part of Saudi Arabia, known for high temperature and humidity, found that concrete is more likely to crack with and without the environmental conditions proposed by [28,31].

Nabil et al. [32] examined substrate bases of concrete (50 × 95 × 365 mm) for plastic shrinkage cracking in an environmental chamber by covering concrete with plastic sheets. The concrete mixes were exposed to a temperature of 55 °C during the first 8 h after casting and 50 °C until the end of the test (24 h). The relative humidity (RH) was about 10%, and the wind speed was 10 km/h during the duration of the test. As expected, it was found that covering concrete with plastic sheets was more efficient in minimizing plastic shrinkage cracking and reducing loss of water compared with non-covering. Almutairi et al. [33] did a survey to determine the causes of all early-age cracking in concrete structures in Kuwait city and concluded that the environmental conditions were the main reason for most the concrete cracking, but also high concrete temperature. It was recommended to prevent early-age cracking. The concrete temperature should be controlled by adding ice to the mixing water.

Almusallam et al. [29] and Safiuddin et al. [34] found that plastic shrinkage cracks increase with an increase in the water/cement ratio and content of fine aggregate. Sayahi and Hedlund et al. [26] reported that micro-settlement cracks also occur on the surface of the concrete. Sulakshna et al. [35] examined a Poly Carboxylate Ether (PCE) as shrinkage reducing admixture (SRA) to self-compacting concrete of *w*/*c* ratio of about 0.45, with encouraging results.

Zhang and Xiao [36] investigated the effect of recycled sand as fine aggregate for 3D-printed mortar on plastic shrinkage cracks. The replacement ratios tested were at 25%, 50%, 75%, and 100% of natural sand, and we had to use high *w*/*c* (0.6) due to the high-water absorption of the recycled sand. The results showed that increased replacement ratios of recycled sand mortar resulted in increased plastic shrinkage cracking. Cohen et al. [37] found that the increase in fine content in concrete (such as fly ash, silica fume, slag, etc.) is not favorable in relation to micro and plastic shrinkage cracking. Lofgren and Esping et al. [38] came to the same conclusion when using silica fume. Zhao et al. [39] examined the influence of clay minerals in manufactured sand and found that as clay lowers the permeability, it also reduces the plastic tensile strength, which leads to an increase in plastic shrinkage cracking.

In conclusion, plastic shrinkage cracking is likely to worsen with climate change, and further work is needed to understand both how the environment and mitigation measures affect its development.

### 1.1. Problem Statement

The increase in harsh environmental conditions created by climate change can significantly affect concrete durability, in particular, due to their impact on plastic shrinkage. Hence, there is a need to investigate such conditions and their impact on concrete with various compositions, as well as possible mitigation measures.

### 1.2. Selection of Environmental Conditions

Saudi Arabia has been selected as a case study region in this investigation as it typifies extremely hot environments, with a drier climate in the northern and central areas and a wetter climate in the southern, western, and eastern areas. Despite the significant variation in humidity, most of the countries in the Middle East share the same high air temperatures [33].

Hasanain et al. [40] tested the effects of hot weather on the evaporation rate of concrete slabs in external daytime environmental conditions in Jeddah, the biggest western city in Saudi Arabia, known for its hot-wet weather due to its location next to the Red Sea. The results showed that casting at noon or afternoon had a higher evaporation rate compared to casting early in the morning. The minimum evaporation rate was recorded when casting in the morning and occurred 3–5 h after casting when the concrete had partly been set.

#### Selected Environments

**Figure 2:**
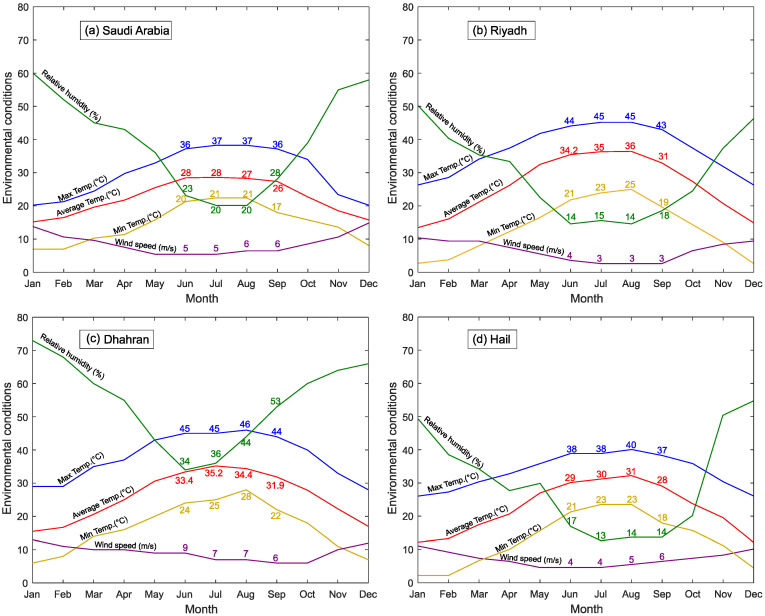
Selected environments climate graphs [41].

Three cities in Saudi Arabia were chosen to evaluate the effect of concreting under severe hot and dry weather conditions. Figure 2 shows the “Climate Graphs” for Saudi Arabia and the three case study cities, including average, minimum and maximum air temperature; relative humidity; and wind speed [41]. While the overall conditions for Saudi Arabia reflect well the ASTM C1579 [28] conditions, the specific environments of each city depart from these climatic settings.

(a) Riyadh

The capital of Saudi Arabia, Riyadh city, is located in the middle of Saudi Arabia. Temperatures are high in the summer, and the relative humidity is very low in both winter and summer (see Figure 2b). During the months of June to September, the air temperature, relative humidity, and wind speed are at the levels that increase the possibility of plastic shrinkage cracks, as anticipated by ASTM C1579 [28], and concreting at temperatures around 45 °C is not uncommon. This (high) temperature level will be examined in this study, as well as the effect of a lower average wind speed of 3 m/s.

Arafah et al. [42] investigated the effects of hot weather on the strength of concrete cast in Riyadh during summer, with target temperatures of 45–48 °C. The high temperature caused high bleeding. Concrete cubes, covered with burlap and cured by water sprinkling two times a day, resulted in concrete strength lower than the ACI 305 [43] general requirement. Khan and Abbas et al. [44] reported the influence of hot weather conditions in Riyadh city on concrete made with cement and cement replacements silica fume (SF) and fly ash (FA). It was observed that the initial concrete strength tends to increase with moist curing, but that had insignificant effects on long-term strength. In addition, shrinkage cracks were observed on the surface even though moist curing was applied.

(b) Dhahran

Dhahran city, on the eastern side of Saudi Arabia, is one of the hottest cities in Saudi Arabia and the Middle East region, and its climate is characterized by high humidity levels in both winter and summer (see Figure 2c) as it is located by the Gulf Sea. Although high humidity is not expected to affect plastic shrinkage adversely, the high wind speed of 7 m/s typical of this region is, and this wind speed is considered as “high” in this study.

Al-Gahtani [45] conducted a study in Dhahran examining the impact of curing methods on concrete specimens made with cement and cement replacements (SF and FA). The specimens were cured by covering them with wet burlaps or applying curing compounds. The results showed that specimens cured under wet burlap showed better strength development than those treated with curing compounds. The impact of hot temperature on plastic and drying shrinkage, however, was very significant for both curing methods. Nasir and Syed [46] also studied the influence of the water-cement ratio (in the range of 0.3 to 0.45) at different air temperatures (25 to 45 °C) and different curing methods such as concrete covering ponding, and use of curing compounds. All curing methods showed a positive impact on the concrete strength parameters at all *w*/*c* ratios and temperatures used in the study. A high evaporation rate was observed for the plain concrete (without special curing), and this was attributed to the high heat evolution, which in turn had a negative impact on the overall shrinkage behavior.

(c) Hail

Known as one of the coolest cities in winter in the country, Hail is one of the northern cities in Saudi Arabia. Few studies have examined concreting in this region, but while the relative humidity in this part of the country is relatively low during the months of June to September, the combination of environmental conditions experienced in this area is likely to affect plastic shrinkage [28,31,43] recommendations. The lower temperature of 28 °C will be included in the study (see Figure 2d).

Due to the different levels of relative humidity in the selected case study regions, the relative humidity was kept around 20%, which is close to the minimum over the summer for Saudi Arabia (see Figure 2a).

### 1.3. Significance of Research

This paper examines the impact of various environmental conditions (high, mid, and low—wind speed, air temperature, and relative humidity) on the plastic shrinkage performance of concrete made with different *w*/*c* ratios. The addition of different dosages of recycled tire steel fiber (RTSF) is also examined as a plastic shrinkage mitigation measure. The results will help improve concrete performance in hot regions such as the Middle East and in more extreme environments created by climate change and will reduce the use of natural and virgin materials by reusing tire steel fiber to control early-age cracking in concrete structures.

## 2. Materials

### 2.1. Concrete and Specimen Preparations

As the likelihood of plastic shrinkage cracking increases with high cement content, a relatively high cement content was selected [47]. The cement (CEMII 42.5) content was 335 kg/m^3^ with a *w*/*c* of 0.55 (for the reference mix) superplasticizer (Sika ViscoCrete 30HE; Sika AG, Baar, Switzerland) was used at a dosage of 1.5 lt/m^3^. Two sizes of gravel (river round gravel) were used, 491 kg/m^3^ of 5–10 mm, and 532 kg/m^3^ of 10–14 mm. The fine aggregate (river-round sand) was 847 km/m^3^. RTSF was used at different proportions (0, 30 kg/m^3^, and 40 kg/m^3^).

The water-cement ratios that were used in this study include 0.5, 0.55, and 0.6 as Low, Mid, and High ratios, respectively.

Concrete mixing and specimen casting were carried out according to [28,48]. The mixes used with the various key parameters are shown in Table 1. A slump test was made to control the workability of all mixes (RTSFC and PC) to reach the target of the slump test, which is more than 100 ± 10 mm.

### 2.2. Recycled Tire Steel Fibre (RTSF)

Waste disposal is becoming a critical environmental pollution issue worldwide. According to Mohajerani et al. [49], billions of tires are replaced every year around the world, while half of them are disposed of by burning or landfilling. Nowadays, there is an increasing interest in the use of secondary raw materials in the construction field of civil engineering [50,51]. Recycled tire steel fibers (RTSF), produced from waste tires, have been used as a substitute for manufactured steel fibers (MSF) and were shown to improve the performance of concrete and enhance its cracking resistance due to shrinkage and structural loads [52,53,54,55].

Hu et al. [56] showed that RTSF could improve the splitting and flexural strength of concrete and result in comparable (or better) performance to MSF. Baricevic et al. [57] examined the effect of using blends of recycled tire steel and manufactured steel fibers, and the results showed a positive impact in delaying the development of drying shrinkage cracks. Graeff et al. [14] examined the performance of RTSF concrete under cyclic loading and showed that the addition of RTSF can improve the fatigue behavior of concrete and help restrain micro-cracks.

The advantages of using RTSF instead of MSF in concrete are not just in terms of overall performance but also in terms of economic and environmental benefits, as RTSF has lower greenhouse gas emissions and cost compared to MSF [58,59,60]. Moreover, Mastali et al. [61] estimated that the use of 1.5% (by volume) of RTSF can make up about 35% of the total cost, while this increase to more than 50% when using MSF. According to the same study, the use of RTSF also contributed to lower carbon emissions from 40% (for 1.5% of MSF) to 15% (for 1.5% of RTSF).

**Figure 3:**
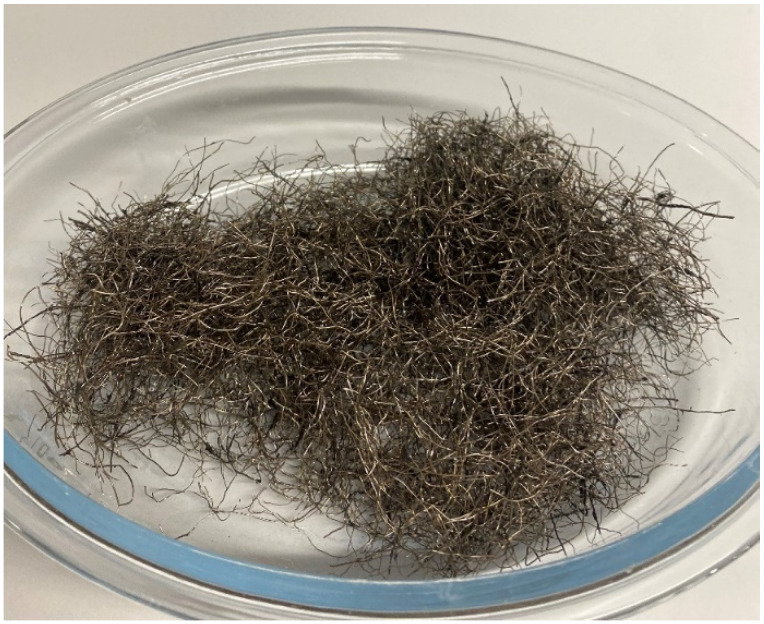
RTSF.

**Figure 4:**
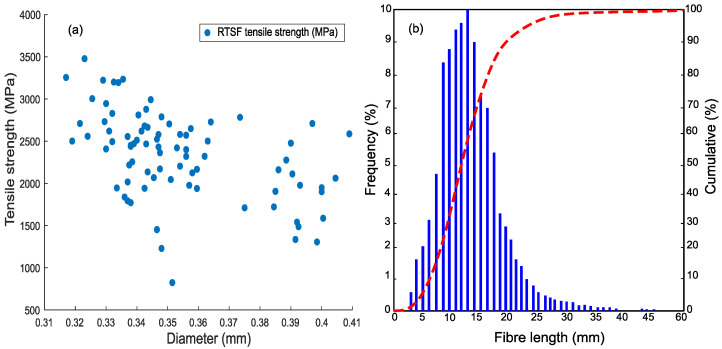
(**a**) Tensile strength; and (**b**) RTSF length distributions [64].

The authors showed in previous work by Alshammari et al. [62] that RTSF of 30 kg/m^3^ by volume can stop plastic shrinkage cracks. That same amount is used in high, mid, and low environmental conditions and increased to 40 kg/m^3^ if cracks develop. RTSF (see Figure 3) has different lengths and diameters with an average tensile strength of 2380 MPa (SD = 166 MPa), as shown in Figure 4a as tested in the lab for more than 100 samples of RTSF. The tensile strength test was carried out according to ISO 6892-1 [63]. The length distribution of RTSF, which was determined by an automated optical method [64], is shown in Figure 4b.

## 3. Methodology

### 3.1. Compressive Strength

The 28-day compressive strength of all mixes were determined from 100 mm cubes tested using a servo-hydraulic universal testing machine according to [65]. For each mix, half of the cubes were initially stored in the environmental chamber under the different environmental conditions applied during the plastic shrinkage test. The other half was stored in normal lab conditions 20 ± 2 °C.

### 3.2. Evaporation Rate

Two aluminum pans filled with water were placed inside the chamber. Each water pan rested on a scale to quantify the evaporation rate at time intervals of 30 min, as recommended by [28]. The evaporation rate at each time interval was determined by (Equation (1)), and if the average evaporation rate was less than 1.0 kg/m^2^/h, the test was rejected [28].

(1) $$E=\frac{M2-M1}{water\;surface\;area\;of\;the\;pan\times \left(T2-T1\right)}$$

where, *E*: Evaporation rate, kg/m^2^/h, *M*2 − *M*1: the mass loss between successive weighings, g. and *T*2 − *T*1: the time interval between successive weighings, h.

### 3.3. Plastic Shrinkage Test

**Figure 5:**
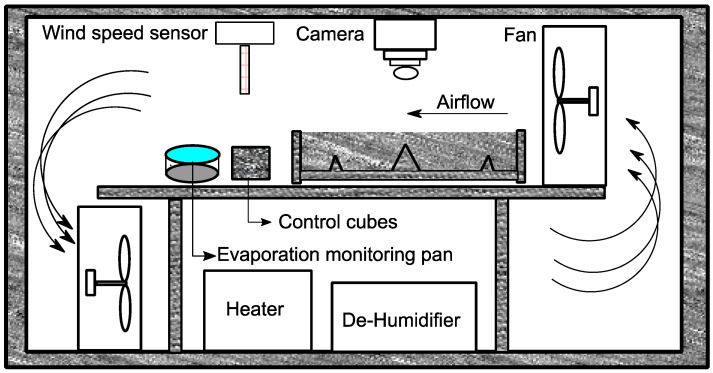
Schematic section of the chamber.

The test was carried out on fresh/plastic concrete in accordance with ASTM C1579 [28] and ACI Committee 305 [31] and typically lasted 6 h. Two concrete slabs were tested in parallel in the chamber (see Figure 5).

At the end of the test (after 6 h), the doors of the chamber were left open, and the slabs were covered with a plastic sheet and left undisturbed for an additional 18 h. The cracks were then measured 24 h again after casting.

### 3.4. Measurement of the Cracks

The method used to measure the cracks followed the recommendations of ASTM C1579 [28], coupled with digital image processing (DIP). Digital photographs of the surface of the specimens were taken at regular intervals during the test and at 24 h from casting and subsequently processed in MATLAB to determine the length, width, and area of the plastic shrinkage cracks [5]. Moreover, at 24 h after the concrete was cracked, a conventional manual optical method was used to measure the average crack width at more than 25 points of the crack by using a millimeter steel ruler and compared to the DIP. The crack reduction ratio (CRR) was determined by using Equation (2), as recommended in [28].

(2) $$\mathrm{CRR}\;=\left[1-\frac{\mathrm{Average}\;\mathrm{Crack}\;\mathrm{Width}\;\mathrm{of}\;\mathrm{Fibre}\;\mathrm{Reinforced}\;\mathrm{Concrete}\;\mathrm{Mixture}}{\mathrm{Average}\;\mathrm{Crack}\;\mathrm{Width}\;\mathrm{of}\;\mathrm{Fibre}\;\mathrm{Control}\;\mathrm{Concrete}\;\mathrm{Mixture}}\right]\times 100\%$$

## 4. Examined Environmental Conditions

**Figure 6:**
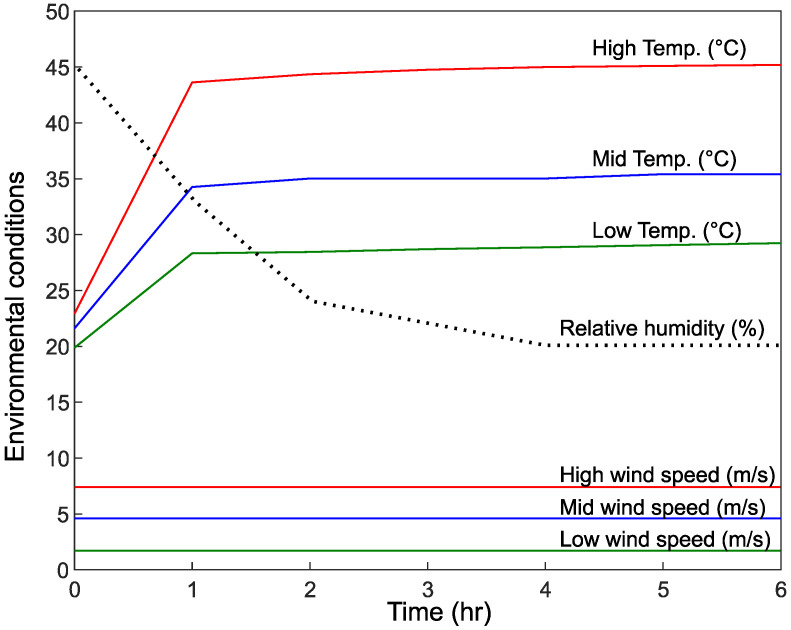
Examined sets of environmental conditions.

Figure 6 shows all the environmental conditions examined in this study in terms of temperature and wind speed values. The mid values correspond to those also recommended in ASTM C1579 [28] and include a wind speed of 4.7 m/s, a temperature of 36 ± 3 °C, and relative humidity of 30 ± 10%. The low temperature of 28 °C was selected as it corresponds to the minimum temperature during the day in the three case study areas (Riyadh, Dhahran, and Hail), while the high temperature of 45 °C corresponds to the higher temperature experienced in the region during the hot months.

The low wind speed of 3 m/s and the high wind speed of 7 m/s corresponds to the average wind speeds experienced in the summer in Riyadh and Dhahran, respectively.

## 5. Experimental Results and Discussion

### 5.1. Compressive Strength

**Figure 7:**
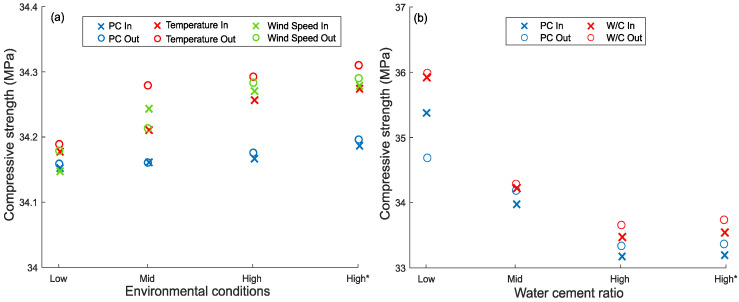
Compressive strength of PC and FRC at 28 days of curing for cubes inside and outside the chamber. * higher content of RTSF (40 kg/m^3^).

The 28-day compressive strength of the control specimens (PC) and SFRC reinforced specimens was determined on cubes that were kept both insides (In) and outside (Out) the chamber during the plastic shrinkage tests, and the corresponding values are summarized in Figure 7.

The results do not show any significant influence of temperature and wind speed on the compressive strength of the cubes that were kept inside the chamber (see Figure 7a). As expected, a slight increase in compressive strength can be seen with increased fiber content [66,67,68].

Naturally, concrete strength increases with the reduction in the water-cement ratio (see Figure 7b) in line with other works [69,70,71]. This can be attributed to the closer spacing between the cement particles, which results in a denser and stronger cement paste [72].

### 5.2. Evaporation Rate

**Figure 8:**
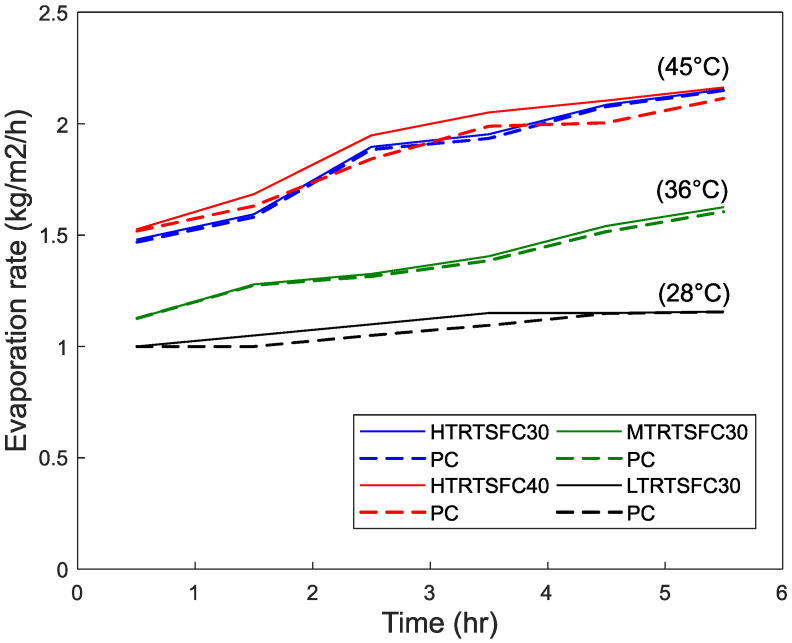
Effect of temperature on evaporation rates (wind speed = 4.7 m/s).

**Figure 9:**
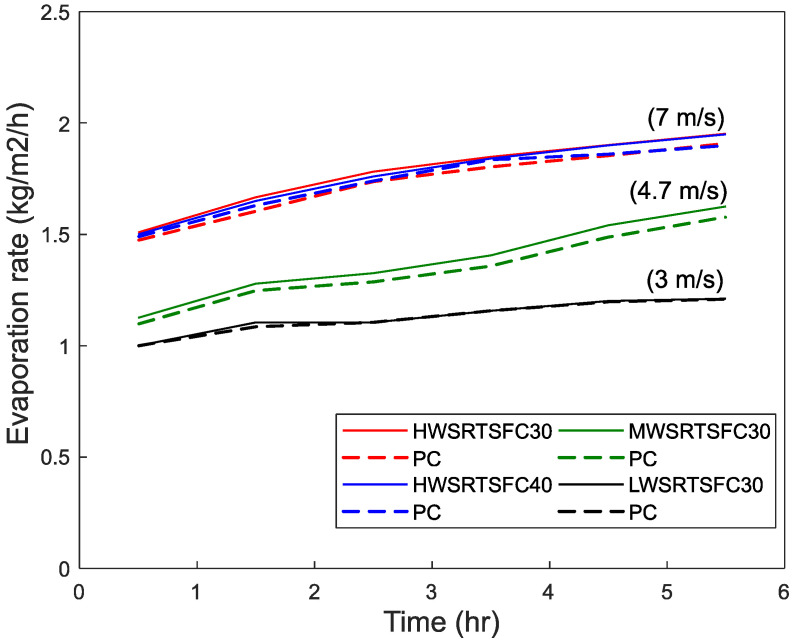
Effect of wind speed on evaporation rates (T = 36 C).

**Figure 10:**
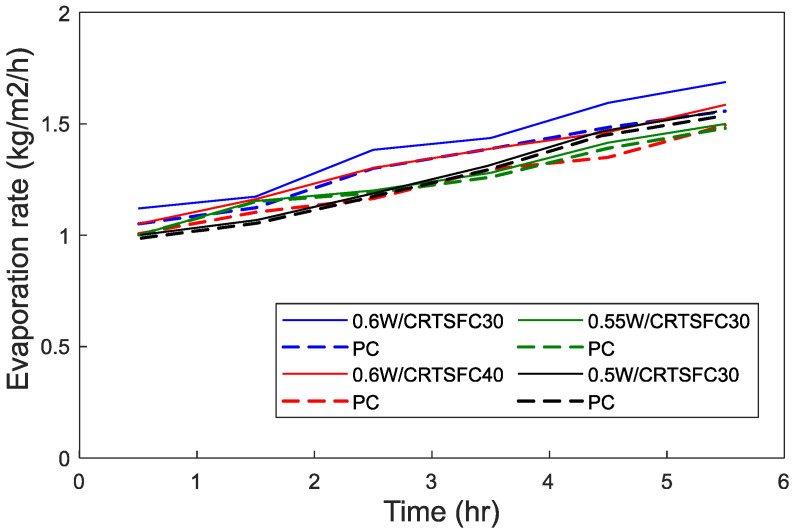
Effect of water cement ratio on evaporation rates (T = 36 °C, Wind speed = 4.7 m/s).

Figure 8, Figure 9 and Figure 10 show the evaporation rates determined for the different environmental conditions. As recommended in ASTM C1579 [28], the evaporation rate should be more than 1 kg/m^2^/h, or the test should be rejected. It can be seen that all the environmental conditions exceeded that rate and achieved the minimum value even in the low environmental conditions. As expected, in general, the evaporation rates increase with time, as bleeding gathers momentum and concrete temperature increases with the heat of hydration and then slows down towards the end of the test as bleeding slows down with setting.

The evaporation rate increases with wind speed and temperature and nearly doubles from Low to High conditions. This can be attributed to the higher bleeding induced by temperature and wind, as also reported by others [25,27,73,74,75,76,77,78].

The evaporation rates for low, mid, and high water-cement ratio mixtures are shown in Figure 10. It can be seen that higher water-cement ratio somewhat increases the evaporation rate, as more water is available to bleed. Holt and Leivo [79] and Kayondo et al. [26] had a similar finding which led to plastic shrinkage cracks appearing faster on the surface of the concrete.

### 5.3. Crack Measurements and Results

**Figure 11:**
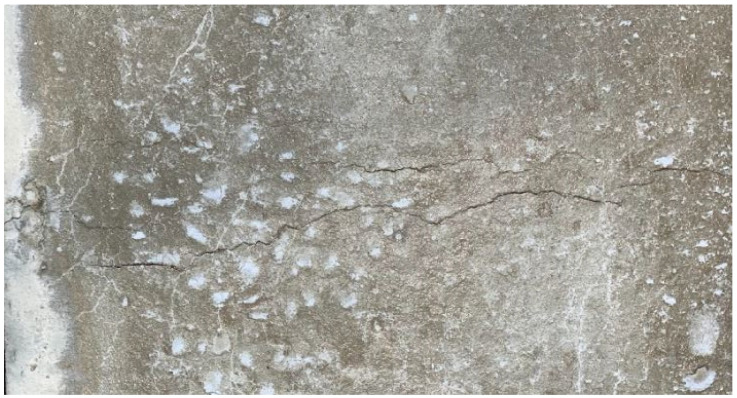
Typical crack pattern in plain concrete (PC) specimens of high temperature examined at 24 h.

**Figure 12:**
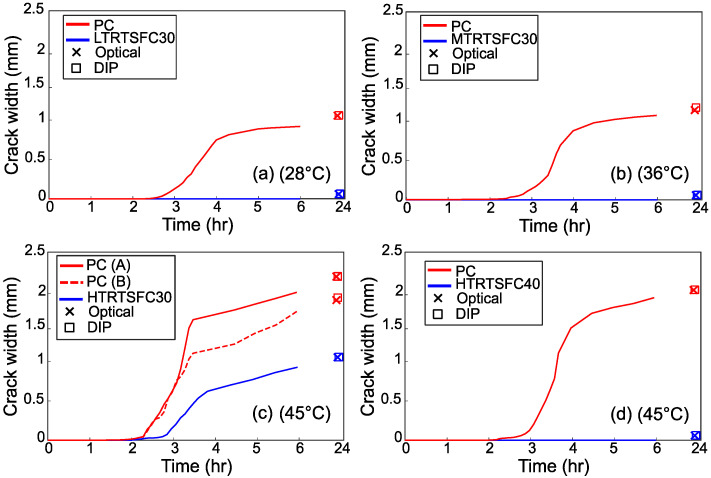
Crack evolution for the specimens subjected to the examined temperatures.

**Figure 13:**
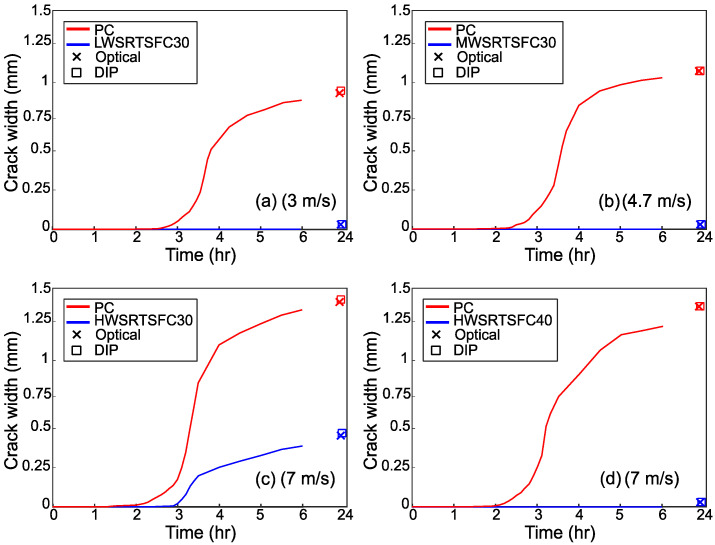
Crack evolution for the specimens subjected to the examined wind speeds.

**Figure 14:**
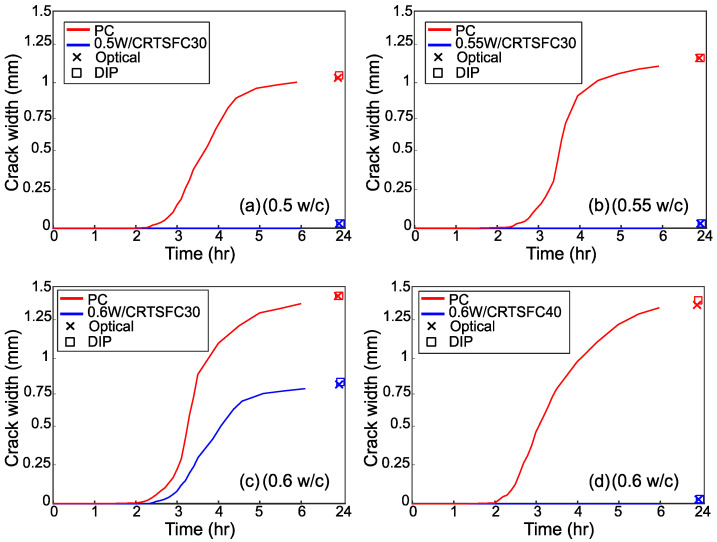
Crack evolution for the specimens manufactured using different *w*/*c* ratios.

For crack measurement, a DIP system was used in which two cameras were placed above the slabs, with each camera taking images every 10 min during the test. The images were subsequently analyzed using MATLAB to measure an average crack (width). A typical photo of the crack for the plain mix is shown below (Figure 11):

Figure 12, Figure 13 and Figure 14 show the evolution of the cracks for the three variable parameters: temperature, wind speed, and *w*/*c* ratio, respectively. The graphs also show the crack widths at 24 h measured both with DIP and a conventional manual optical device. The crack widths obtained for the two methods of measurement are almost identical, confirming that DIP works well. The mean difference between the two measurements was 0.113 mm.

Figure 12 shows that with a temperature increase, cracking starts earlier, and the eventual crack width is wider by almost 100% when comparing Low and High temperatures. The use of 30 kg/m^3^ of RTSF seems to prevent cracking completely for the Low and Medium temperatures. However, though the addition of RTSF delays and helps control the crack, cracking still develops at High temperatures. For the more extreme temperature tested in this study, the increase in fiber dosage to 40 kg/m^3^ seems to prevent plastic cracking.

Similarly, to what was observed for increasing temperature values, increasing wind speed causes earlier cracking to develop and eventually leads to larger crack widths. Interestingly, both the Low wind speed and Low-temperature environments, which are below the ASTM C1579 [28] recommendations, led to cracks (see Figure 13).

Again, the use of RTSF at a dosage of 30 kg/m^3^ prevented cracking in the specimens subjected to the Low and Medium wind speeds only, whilst 40 kg/m^3^ of RTSF was effective in preventing cracking at High wind speeds.

Figure 14 shows the crack width evolution for different *w*/*c* ratios. As expected, the use of lower *w*/*c* ratios delayed the crack initiation and development. As with the previous results, the addition of 30 kg/m^3^ of RTSF was not sufficient to prevent cracking in the specimens with a high water-cement ratio, but 40 kg/m^3^ of RTSF prevented the formation of cracks completely.

### 5.4. Influence of Environmental Conditions on Evaporation Rate and Cracking

**Figure 15:**
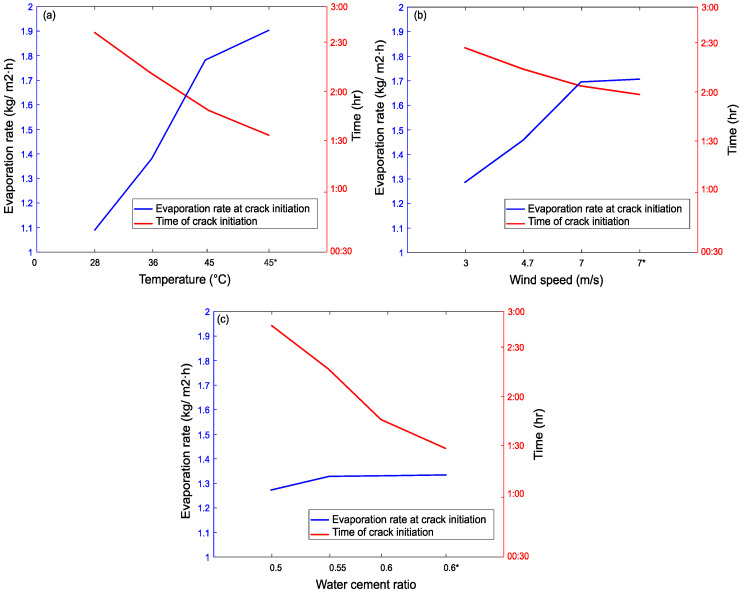
Effect of (**a**) temperature; (**b**) wind speed; and (**c**) *w*/*c* ratio on evaporation rate at crack initiation and time of crack initiation. * higher content of RTSF (40 kg/m^3^).

The effect of the three parameters examined in this study on the evaporation rate at crack initiation and time of crack initiation is shown in Figure 15.

As expected, for all mixes, cracking starts earlier, and the evaporation rate is higher with increasing temperature, wind, and *w*/*c* ratio, and the final cracks are wider. The plain mid-temperature concrete cracked just before the second hour, as expected by [28].

Cracks develop even at the low temperature of 28 °C but crack initiation is delayed by about an hour when compared to specimens exposed to the highest examined temperature. Though surface temperature cannot be changed easily, to reduce plastic shrinkage cracking, the concrete temperature can be cooled down by using cooler materials, including iced water.

In the case of high air temperature (see Figure 11), it was noticed that the plain concrete developed two cracks on the surface, which could adversely impact the durability of the concrete.

At low wind speeds (3 m/s), the evaporation rate reduces but the plain concrete cracks only a bit later and a bit less wide. Hence, at the ranges used, wind speed seems to play a less important role than temperature.

The *w*/*c* ratio seems to influence the evaporation rate the least, compared to temperature and wind. However, the *w*/*c* ratio has a big influence on the time of crack initiation. This was attributed by Topçu, and Elgün [80] to a high bleed and evaporation rate, though the results contradict the higher evaporation rate. Hence, this study concludes that high *w*/*c* ratios only accelerate the initiation of high cracking due to the earlier and higher bleed rate.

### 5.5. Crack Reduction Ratio (CRR)

**Figure 16:**
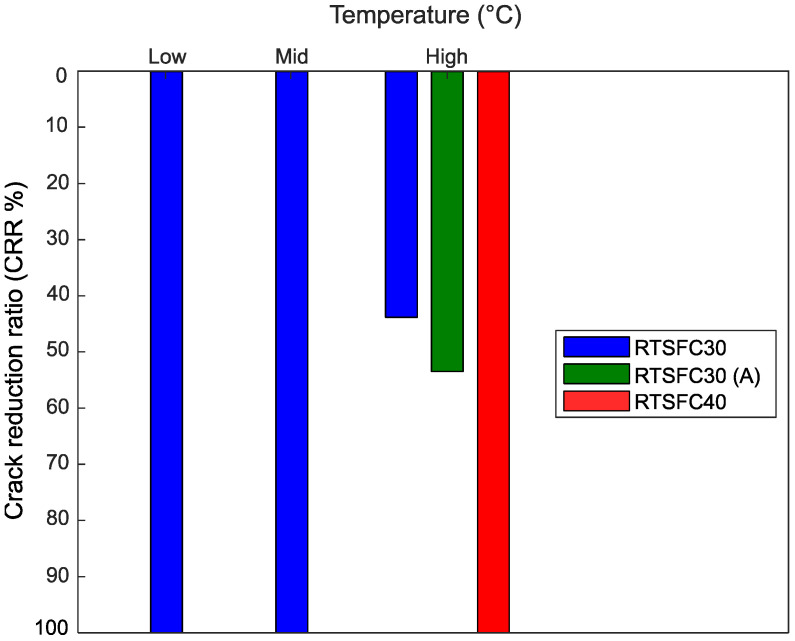
Crack reduction ratio for all temperatures.

Crack reduction ratios were determined in this study according to Equation (1). Figure 16 shows these ratios for the different temperatures examined in this study for concrete mixes with 30 kg/m^3^ and 40 kg/m^3^ of RTSF. It can be seen that the addition of 30 kg/m^3^ of RTSF can eliminate the occurrence of plastic shrinkage cracks at low (28 °C) and mid (36 °C) temperatures.

In comparison, Borg et al. [81] examined the use of recycled PET fibers (polyethylene terephthalate) to control plastic shrinkage cracks and found that 1.5% of fiber (by volume) could reduce cracking by up to 57%. Pešić et al. [82] examined the effect of adding recycled high-density polyethylene (HDPE) plastic fibers and found that early-age concrete cracks can be reduced by up to 50% with a fiber volume of 1.25%. Hence, steel fibers appear to be a more robust solution for stopping plastic shrinkage cracks.

At the highest examined temperature of 45 °C (High), the plain concrete developed two surface cracks, and the corresponding CRR rations are represented in Figure 16 by a blue and green (A) bar, showing that the addition of 30 kg/m^3^ of RTSF effectively reduced these cracks by up to 45%, and 55%, respectively. The addition of RTSF at a rate of 40 kg/m^3^ eliminated the cracks completely, and this dosage is recommended for these high temperatures.

Al-Tulaian et al. [83] used recycled plastic fibers (RP) with two lengths (50 and 20 mm) to restrain plastic shrinkage in mortar concrete at high temperatures (45 °C) and found that RP reduced the plastic shrinkage cracks by up to 70% for a volume fraction of 1.5% and fiber length of 50 mm. Hence, steel fibers appear to be the only solution to stop cracks completely at these high temperatures.

**Figure 17:**
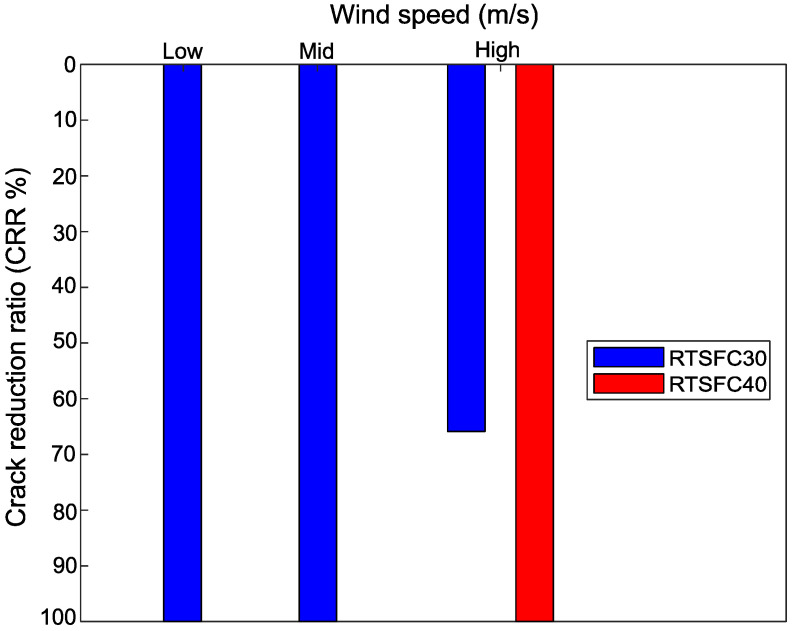
Crack reduction ratio for all wind speeds.

The CRR for the examined values of wind speed is shown in Figure 17. Again, 30 kg/m^3^ of RTSF eliminated cracks at low and mid-wind speeds, while 40 kg/m^3^ of RTSF was necessary to eliminate plastic shrinkage cracking at high wind speeds.

Sirajuddin and Gettu [84] examined the plastic shrinkage behavior of specimens exposed to a wind speed of 4.5 ± 1 m/s and made of concrete comprising cement and fly ash and granulated blast furnace slag and reinforced with different volume fractions of four types of fiber (polypropylene, polyester fibers, polyacrylonitrile and glass fibers). The results showed that increasing the volume fraction of the different types of fibers led to a reduction in cracking by up to 60%.

**Figure 18:**
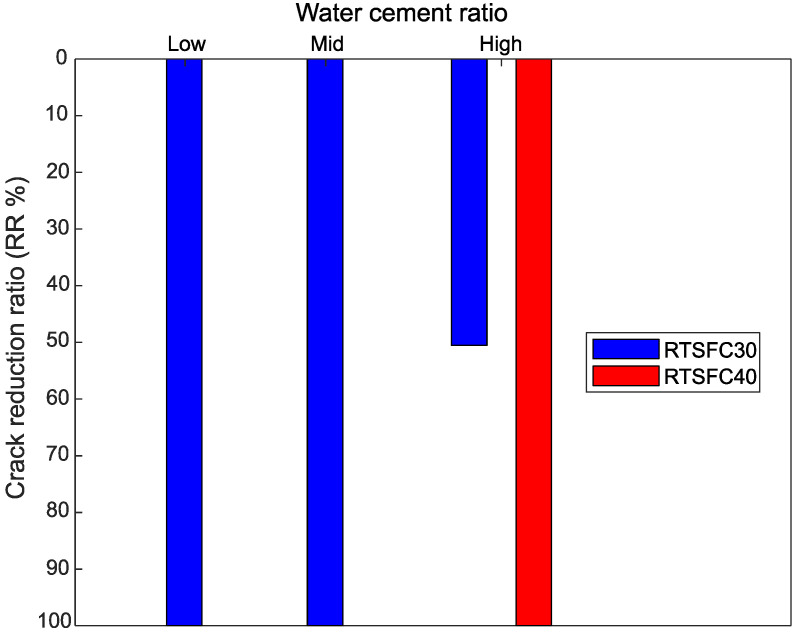
Crack reduction ratio for all water cement ratio.

The CRR for various *w*/*c* ratios is shown in Figure 18. In this case, again, the 30 kg/m^3^ of RTSF eliminated cracks at low and mid *w*/*c* ratios, while 40 kg/m^3^ of RTSF eliminated the cracks at the high *w*/*c* ratio.

Mazzoli et al. [85] examined the impact of adding different types of fibres (polypropylene, polyvinyl alcohol, polyethylene, steel) to restrain plastic shrinkage cracks of concrete with a *w*/*c* ratio of 0.5 and observed that the fibres were effective in reducing the crack width, but the cracks were still visible at the surface of the concrete.

## 6. Conclusions

This paper discusses the impact of low, mid, and high environmental conditions on the plastic shrinkage of concrete. Two *w*/*c* ratios and dosages of RTSF were used to assess their effect in restraining cracking in fresh concrete. From the results, the following conclusions can be drawn:

- Harsher environmental conditions result in earlier and more severe cracking (i.e., larger crack width, length, and area).
- Temperature is the most important parameter in accelerating cracking, whilst wind speed is the least significant.
- The use of higher water/cement ratios increases cracking, primarily as a result of earlier and heavier bleeding.
- The addition of RTSF fibers was found to be effective in controlling plastic shrinkage cracking, with 40 kg/m^3^ eliminating the cracks completely in the harshest of the examined environments.

## Figures and Tables

**Table 1:** Parameters of the study.

Mix ID	Temp. °C	Wind Speed (m/s)	*w*/*c*	RTSF (km/m^3^)
LWSRTSFC30	36	3	0.55	30
MWSRTSFC30	36	4.7	0.55	30
HWSRTSFC30	36	7	0.55	30
HWSRTSFC40	36	7	0.55	40
LTRTSFC30	28	4.7	0.55	30
MTRTSFC30	36	4.7	0.55	30
HTRTSFC30	45	4.7	0.55	30
HTRTSFC40	45	4.7	0.55	40
0.5 W/CRTSFC30	36	4.7	0.50	30
0.55 W/CRTSFC30	36	4.7	0.55	30
0.6 W/CRTSFC30	36	4.7	0.60	30
0.6 W/CRTSFC40	36	4.7	0.60	40

## Data Availability

Not applicable.
